# Multi-sequence magnetic resonance imaging radiomics integrated with clinical features predicts pelvic lymph node metastasis in cervical cancer: a retrospective-prospective cohort study

**DOI:** 10.3389/fmed.2026.1910248

**Published:** 2026-10-12

**Authors:** Wanqing Gong, Xiaoyi Liu, Mengli Wang, Xiaowen Hu, Guangping Yan, Kun Liu, Yongxin Li, Tingting Zhang

**Affiliations:** 1 Department of Radiology, Yichang Central People’s Hospital, Yichang, Hubei, China; 2 Department of Nursing, Yichang Central People’s Hospital, Yichang, Hubei, China; 3 School of Automation and Intelligence, Beijing Jiaotong University, Beijing, China; 4 Department of Thoracic Surgery, Beijing Genertec Aerospace Hospital, Beijing, China

**Keywords:** cervical cancer, deep learning, lymph node metastasis, magnetic resonance imaging, prediction model, radiomics

## Abstract

**Background:**

Pelvic lymph node metastasis is a major determinant of staging, recurrence risk, and treatment selection in cervical cancer, yet conventional magnetic resonance imaging criteria based on nodal size and morphology remain insufficient for occult nodal disease. We developed and temporally validated a multi-sequence magnetic resonance imaging radiomics-clinical fusion framework for pretreatment prediction of pelvic lymph node metastasis.

**Methods:**

This single-center retrospective-prospective cohort study included 200 women with histologically confirmed cervical cancer who underwent pretreatment pelvic magnetic resonance imaging. A retrospective training cohort (*n* = 100) was accrued from January 2018 to December 2022, and a prospective temporal validation cohort (*n* = 100) was accrued from January 2023 to December 2024. Reference standards comprised surgical histopathology in 154 patients and composite clinical-imaging adjudication in 46 patients. Primary tumors were manually segmented on axial T2-weighted imaging and registered to T1-weighted imaging, diffusion-weighted imaging/apparent diffusion coefficient maps, and dynamic contrast-enhanced imaging. An Image Biomarker Standardisation Initiative-compliant radiomics pipeline was used, including resampling, intensity discretization, reproducibility filtering, correlation pruning, and nested feature selection. Clinical, radiomics, clinical-radiomics, and deep fusion models were evaluated in the prospective cohort using discrimination, calibration, decision-curve, and reclassification analyses.

**Results:**

The deep fusion model achieved the best validation discrimination, with an area under the receiver operating characteristic curve of 0.910 (95% confidence interval, 0.845–0.959) and an area under the precision-recall curve of 0.849. At the training-derived threshold, accuracy was 0.84, sensitivity was 0.79, specificity was 0.86, positive predictive value was 0.75, negative predictive value was 0.89, and Brier score was 0.125. Deep fusion outperformed the clinical model (area under the curve, 0.730; mean delta area under the curve, 0.180; *P* < 0.001) and improved reclassification relative to the clinical model.

**Conclusion:**

Multi-sequence magnetic resonance imaging radiomics-clinical fusion improved pretreatment risk stratification for pelvic lymph node metastasis in cervical cancer. External multicenter validation is warranted before clinical implementation. These preliminary single-center findings should be interpreted cautiously and require multicenter external validation before broad clinical implementation.

## Introduction

Cervical cancer remains one of the most common gynecologic malignancies worldwide and continues to impose a substantial clinical burden, particularly in low- and middle-income regions (1, 2). Pelvic lymph node metastasis (LNM) is one of the most important prognostic factors in cervical cancer because it is closely associated with recurrence, distant spread, and survival. Nodal status can materially alter treatment selection between radical surgery, chemoradiotherapy, and extended-field irradiation (3, 4). Accurate pretreatment estimation of pelvic nodal status is therefore essential for risk-adapted management.

Pelvic magnetic resonance imaging (MRI) is central to local staging because it provides excellent soft-tissue contrast for tumor delineation and parametrial assessment (4). However, conventional MRI-based nodal evaluation still relies mainly on size thresholds, shape, central necrosis, or irregular borders, and these criteria have limited sensitivity for micrometastatic disease. Visible nodal morphology is only an indirect surrogate of metastatic potential and may not fully reflect intratumoral heterogeneity, stromal invasion, cellular density, perfusion heterogeneity, or other biological processes that contribute to nodal spread.

Radiomics converts routine images into high-dimensional quantitative phenotypes by extracting first-order, morphologic, and textural descriptors that may capture tumor behavior beyond subjective image interpretation (5, 6). Several MRI-based radiomics studies have reported promising performance for LNM prediction in cervical cancer, including models based on single-sequence MRI, multiparametric MRI, and hybrid clinical-radiomics nomograms (7–13). Nevertheless, methodological limitations remain common, including single-center retrospective design, insufficient temporal or external validation, incomplete reporting of calibration and clinical utility, and inadequate attention to reproducibility and risk of bias (11–16). The present study does not eliminate all of these limitations. Its incremental contribution is a temporally separated prospective evaluation cohort, leakage-controlled multi-sequence fusion, explicit reporting of reference-standard provenance, and evaluation of calibration, clinical utility, and reclassification; single-center sampling and the lack of external multicenter validation remain unresolved.

We therefore developed a leakage-resistant pretreatment prediction framework that integrates multi-sequence MRI radiomics and routinely available clinical variables for pelvic LNM assessment in cervical cancer. We hypothesized that fusing complementary sequence-specific information from T2-weighted imaging, diffusion/apparent diffusion coefficient (ADC) imaging, dynamic contrast-enhanced (DCE) imaging, T1-weighted imaging, and clinical variables would improve discrimination, calibration, and decision-analytic value compared with clinical assessment alone. This manuscript was prepared with attention to the TRIPOD + AI and CLEAR reporting recommendations (14, 16).

## Materials and methods

### Study design and participants

This single-center study was designed as a retrospective-prospective cohort investigation at Yichang Central People's Hospital. The retrospective development cohort comprised consecutive eligible patients examined between January 2018 and December 2022, and the prospective temporal validation cohort comprised consecutively enrolled patients between January 2023 and December 2024. The patient selection and modeling workflow is summarized in Figure 1.

**Figure 1:**
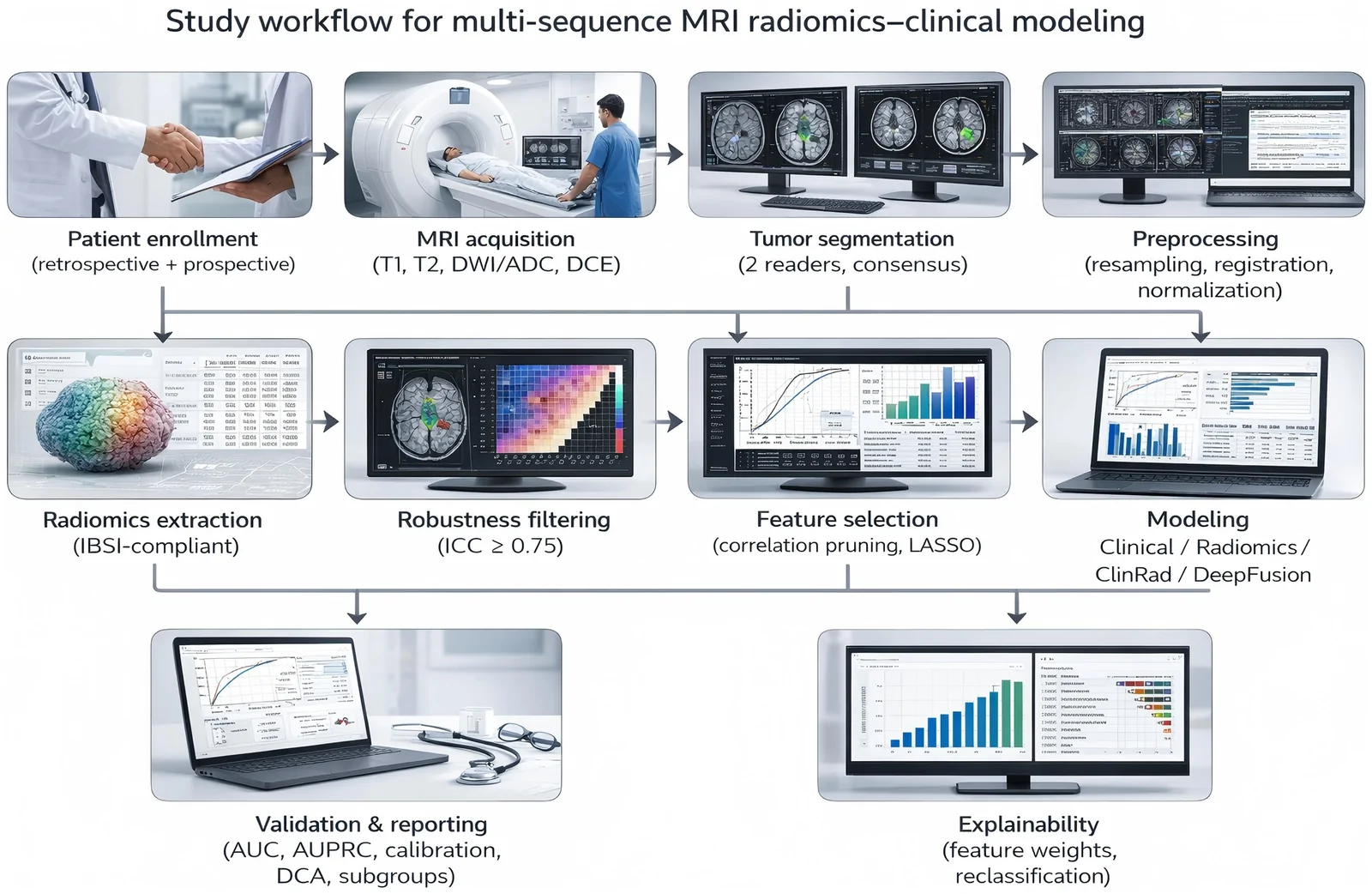
Original schematic workflow for multi-sequence magnetic resonance imaging radiomics-clinical modeling and prospective temporal validation.

Eligibility criteria were: (1) histopathologically confirmed cervical cancer; (2) pretreatment pelvic MRI performed within 2 weeks before surgery or definitive treatment; and (3) availability of a reference standard for pelvic nodal status. Exclusion criteria were prior pelvic radiotherapy or chemotherapy, incomplete MRI sequences, severe motion or susceptibility artifacts that precluded segmentation, incomplete key clinical data, a history of another active pelvic malignancy, or inability to establish nodal outcome status. The study size was determined pragmatically by the number of consecutively eligible patients available during the prespecified accrual periods.

Because cervical cancer is a disease affecting female patients, all included participants were women. Age and clinicopathologic variables were abstracted from the electronic medical record. Candidate clinical predictors were age, maximum tumor diameter, International Federation of Gynecology and Obstetrics (FIGO) stage, histologic subtype, human papillomavirus status, serum squamous cell carcinoma antigen (SCC-Ag), and MRI-reported suspicious pelvic nodes.

### Reference standard and outcome definition

The primary outcome was pelvic LNM status before treatment. LNM positivity was defined according to postoperative histopathology of sampled or dissected pelvic lymph nodes whenever surgical pathology was available. At the patient level, the pathology-based outcome was positive if metastatic cervical carcinoma was identified in any sampled or dissected pelvic node and negative only if all sampled or dissected pelvic nodes were tumor-free.

For patients managed non-surgically under institutional standard care, pelvic nodal status was established using a composite reference standard: image-guided nodal biopsy when clinically feasible; otherwise, multidisciplinary review integrating baseline pelvic MRI, diffusion/apparent diffusion coefficient findings, contrast-enhanced MRI, computed tomography or positron-emission tomography/computed tomography when available, treatment response, serial follow-up imaging for at least 6 months, and clinical records. For clarity, baseline pelvic MRI referred to the same pretreatment examination performed within 2 weeks before surgery or definitive treatment; assessment included nodal size and morphology, high-b-value diffusion signal with the corresponding apparent diffusion coefficient map, and contrast-enhancement pattern. Computed tomography or positron-emission tomography/computed tomography, when available, provided corroborative anatomic or metabolic evidence, while clinical records documented treatment fields, subsequent pathology, and pelvic nodal recurrence. None of these components was considered an independent gold standard. Nodes were classified as metastatic when pathological confirmation was available or when baseline imaging showed typical malignant nodal features with concordant progression or unequivocal response to nodal-directed therapy. Treatment response was considered supportive only when the same baseline-suspicious node showed unequivocal regression of its morphologic, diffusion, enhancement, or metabolic abnormality after nodal-directed therapy. A composite-negative classification required no convincing malignant nodal findings across the available baseline examinations, no subsequent pathologic or imaging evidence of pelvic nodal metastasis, and stable or regressing findings without pelvic nodal recurrence during at least 6 months of follow-up. Stability in nodal diameter alone was insufficient to establish a negative reference standard. Patients whose nodal status could not be adjudicated were excluded before model development. The source of the reference standard was recorded for each patient and categorized as surgical histopathology or composite adjudication to permit transparent assessment of potential verification bias.

### MRI acquisition

All examinations were performed on a 3.0-T pelvic MRI system using the institutional standard cervical cancer protocol. The protocol included axial and sagittal T2-weighted imaging, axial T1-weighted imaging, diffusion-weighted imaging with low and high b values and derived apparent diffusion coefficient maps, and dynamic contrast-enhanced imaging after intravenous gadolinium-based contrast administration.

### Tumor segmentation, registration, and radiomics preprocessing

An Image Biomarker Standardisation Initiative-compliant radiomics workflow was implemented using PyRadiomics (17, 18). Primary tumors were manually segmented three-dimensionally on axial T2-weighted images using ITK-SNAP (version 3.8) (19). The tumor mask was then propagated to the other sequences after rigid or affine co-registration implemented with a validated image-processing workflow (20). Radiologists performing segmentation were blinded to pathologic nodal status and outcome data at the time of contouring. A randomly selected subset of examinations was re-segmented to assess inter- and intra-observer reproducibility.

Before feature extraction, all image volumes were resampled to isotropic 1 × 1 × 1 mm voxels using third-order B-spline interpolation, whereas masks were resampled with nearest-neighbor interpolation. Intensity normalization and fixed-bin-width discretization were applied to reduce the influence of acquisition-scale variability. The initial feature pool included first-order statistics, shape descriptors, and texture matrices, including gray-level co-occurrence matrix, gray-level run-length matrix, gray-level size-zone matrix, gray-level dependence matrix, and neighboring gray-tone difference matrix features.

### Feature robustness and model development

Feature robustness was quantified with the two-way random-effects intraclass correlation coefficient (ICC) for absolute agreement. Only features with an ICC of at least 0.75 were retained for downstream modeling. To reduce redundancy, pairs of features with absolute Spearman correlation coefficients of 0.90 or greater were pruned by retaining the more reproducible or clinically interpretable descriptor. Robustness safeguards were incorporated because MRI radiomics features can be sensitive to segmentation, resampling, and acquisition variation (21).

The remaining standardized features entered a nested feature-selection framework in the training cohort, and least absolute shrinkage and selection operator logistic regression was used to derive a parsimonious radiomics signature. Four model families were constructed. The clinical model incorporated the prespecified clinical and MRI-assessed variables. The radiomics model used the selected radiomics signature alone. The ClinRad model integrated the radiomics signature with the clinical predictors. The deep fusion model combined multi-sequence radiomics information with clinical variables in a fully connected neural-network framework. Hyperparameters were tuned exclusively within the training cohort through nested cross-validation to minimize optimism bias and information leakage.

The deep fusion network used standardized sequence-specific radiomics vectors from T1-weighted, T2-weighted, ADC, and DCE images together with a standardized clinical vector as inputs. Each imaging branch contained one dense layer (16 neurons, rectified linear-unit activation) followed by batch normalization and 30% dropout; branch outputs were concatenated with the clinical vector and passed through two dense layers (32 and 16 neurons, rectified linear-unit activation) with L2 weight decay (lambda=1 × 10^−4) and dropout before a single sigmoid output node. Models were trained with binary cross-entropy using the Adam optimizer (learning rate=1 × 10^−3), a batch size of 16, inverse-frequency class weights, and a maximum of 200 epochs with early stopping if validation loss did not improve for 20 epochs. Hyperparameters were selected only within the nested training-cohort cross-validation loop, and no validation-cohort information was used for feature selection, model tuning, threshold selection, or recalibration.

### Missing data and statistical analysis

Cases with incomplete core clinical predictors or incomplete MRI sequences were excluded before model development; therefore, no statistical imputation was performed for the analytic cohort. All statistical tests were two-sided, and *P* values less than 0.05 were considered statistically significant. Methodological safeguards were selected to address common risk-of-bias concerns for prediction-model studies (22).

Model performance was evaluated in the prospective validation cohort using discrimination, including area under the receiver operating characteristic curve (AUC) with 95% confidence intervals (CIs) and area under the precision-recall curve (AUPRC); threshold-dependent metrics, including accuracy, sensitivity, specificity, positive predictive value (PPV), and negative predictive value (NPV); calibration, including Brier score, calibration intercept, calibration slope, and expected calibration error; and clinical utility through decision-curve analysis (23–27). Binary operating thresholds were determined in the training cohort by maximizing the Youden index and were then transferred unchanged to the validation cohort. Incremental value over the clinical baseline was assessed with bootstrap-based AUC comparison and reclassification analyses using categorical net reclassification improvement (NRI) and integrated discrimination improvement (IDI) (28–30). For reclassification analyses, predicted probabilities were grouped using prespecified cutoffs of 0.20 and 0.40 to define low-, intermediate-, and high-risk categories. No model updating or recalibration was performed in the validation cohort. To characterize potential verification bias, the proportion of LNM-positive patients was compared between the pathology-based and composite-reference groups using Fisher's exact test.

### Ethics approval and consent to participate

The study was conducted in accordance with the Declaration of Helsinki and its subsequent amendments. The study was reviewed by the Ethics Committee of Yichang Central People's Hospital (ethics opinion No. 2024-146-02). Written informed consent was obtained from all participants in the prospective cohort, and the requirement for individual informed consent for the retrospective cohort was waived by the ethics committee. The study was observational and did not assign interventions; clinical trial registration was therefore not applicable.

## Results

### Patient characteristics

A total of 200 eligible patients were included, with 100 patients allocated to the retrospective training cohort and 100 patients allocated to the prospective validation cohort. Pelvic LNM was confirmed in 36 of 100 patients (36.0%) in the training cohort and 34 of 100 patients (34.0%) in the validation cohort. Surgical histopathology served as the reference standard in 154 patients (training, 80; validation, 74), whereas composite adjudication was used in 46 patients (training, 20; validation, 26). The corresponding LNM-positive counts were 28 of 80 and 22 of 74 in pathology-based cases and 8 of 20 and 12 of 26 in composite-reference cases for the training and validation cohorts, respectively. The two cohorts were broadly comparable with respect to age, tumor size, FIGO stage distribution, histology, human papillomavirus status, SCC-Ag concentration, MRI-reported nodal suspicion, and LNM prevalence (Table 1). These findings support the use of the prospective cohort as a temporal validation dataset rather than a substantially different clinical population. Across the full cohort, LNM was present in 50 of 154 pathology-referenced patients (32.5%) and 20 of 46 composite-referenced patients (43.5%); the difference was not statistically significant (Fisher's exact *P* = 0.217). This descriptive comparison was not considered evidence that the two reference standards were equivalent.

**Table 1:** Baseline characteristics of the development and prospective validation cohorts.

Characteristic	Training (*n* = 100)	Validation (*n* = 100)	*P* value
Age, years	49.2 +/- 10.9	48.6 +/- 10.6	0.68
Tumor size, cm	4.2 +/- 1.65	4.1 +/- 1.70	0.77
FIGO stage, n	IB1-IB2: 56; IIA: 18; IIB: 26	IB1-IB2: 58; IIA: 16; IIB: 26	0.94
Histology, n	Squamous: 78; adenocarcinoma: 22	Squamous: 76; adenocarcinoma: 24	0.78
HPV positive, *n* (%)	86 (86.0)	84 (84.0)	0.72
SCC-Ag, ng/mL	4.8 [2.6–8.5]	4.6 [2.5–8.0]	0.61
MRI suspicious nodes, *n* (%)	28 (28.0)	30 (30.0)	0.69
Reference standard, n	Pathology: 80; composite: 20	Pathology: 74; composite: 26	0.31
Pelvic LNM positive, *n* (%)	36 (36.0)	34 (34.0)	0.78

Data are presented as mean +/- standard deviation, median [interquartile range], or *n* (%). FIGO, International Federation of Gynecology and Obstetrics; HPV, human papillomavirus; LNM, lymph node metastasis; MRI, magnetic resonance imaging; SCC-Ag, squamous cell carcinoma antigen.

### Feature robustness and radiomics signature

After reproducibility filtering, only robust features with ICC values of at least 0.75 were retained. Subsequent redundancy reduction and nested least absolute shrinkage and selection operator modeling yielded a compact 12-feature signature spanning first-order, shape, and texture classes across T2-weighted, ADC, DCE, and T1-weighted sequences (Table 2). Selected features included descriptors of intratumoral heterogeneity and morphologic compactness, such as gray-level co-occurrence matrix correlation, gray-level co-occurrence matrix contrast, gray-level size-zone matrix zone variance, gray-level run-length matrix run variance, and shape sphericity.

**Table 2:** Selected radiomics signature and summary statistics in the validation cohort.

Feature	Seq.	Class	ICC	Std. coeff.	LNM-	LNM+	Direction
original_glcm_Correlation	ADC	GLCM	0.91	0.238	−0.54 +/−0.90	0.57 +/−0.92	Higher risk
original_glszm_LargeAreaEmphasis	T2	GLSZM	0.79	−0.048	0.52 +/−0.92	−0.66 +/−0.83	Lower risk
original_glrlm_LongRunHighGrayLevelEmphasis	DCE	GLRLM	0.92	−0.133	0.41 +/−0.87	−0.64 +/−0.93	Lower risk
original_firstorder_Kurtosis	T2	First-order	0.90	0.162	−0.42 +/−0.81	0.54 +/−0.93	Higher risk
original_glcm_Imc1	ADC	GLCM	0.92	0.289	−0.42 +/−0.83	0.73 +/−0.88	Higher risk
original_firstorder_Entropy	DCE	First-order	0.82	−0.071	0.46 +/- 0.89	−0.57 +/−0.83	Lower risk
original_ngtdm_Coarseness	T1	NGTDM	0.80	−0.053	0.42 +/−0.87	−0.65 +/−1.02	Lower risk
original_shape_Sphericity	T2	Shape	0.90	0.240	−0.32 +/−0.86	0.51 +/−1.09	Higher risk
original_glcm_Contrast	ADC	GLCM	0.93	−0.305	0.49 +/−0.85	−0.64 +/−0.86	Lower risk
original_glszm_ZoneVariance	DCE	GLSZM	0.96	−0.530	0.38 +/−0.83	−0.59 +/- 0.89	Lower risk
original_glrlm_RunVariance	T2	GLRLM	0.95	0.138	−0.38 +/−0.90	0.58 +/−0.76	Higher risk
original_firstorder_10Percentile	ADC	First-order	0.83	−0.235	0.37 +/−0.88	−0.63 +/−0.84	Lower risk

ADC, apparent diffusion coefficient; DCE, dynamic contrast-enhanced magnetic resonance imaging; GLCM, gray-level co-occurrence matrix; GLRLM, gray-level run-length matrix; GLSZM, gray-level size-zone matrix; ICC, intraclass correlation coefficient; Imc1, informational measure of correlation 1; LNM, lymph node metastasis; NGTDM, neighboring gray-tone difference matrix; Seq., sequence; Std. coeff., standardized coefficient.

The sequence composition of the final signature suggested that ADC and DCE features contributed complementary information beyond morphologic T2-weighted descriptors alone.

### Model discrimination and classification performance

The performance of all candidate models is summarized in Table 3 and Figure 2. In internal cross-validation, the deep fusion model achieved the highest mean AUC (0.91 +/- 0.03), followed by the ClinRad model (0.89 +/- 0.04) and the radiomics model (0.87 +/- 0.04). This ranking was preserved in prospective validation. The deep fusion model demonstrated the best discrimination with an AUC of 0.910 (95% CI, 0.845−0.959) and an AUPRC of 0.849, whereas the ClinRad model reached an AUC of 0.880 (95% CI, 0.801−0.944). Among the single-sequence radiomics models, ADC performed best (AUC, 0.810; 95% CI, 0.715−0.895), followed by T2-weighted imaging, DCE, and T1-weighted imaging.

**Table 3:** Model performance in internal cross-validation and prospective validation.

Model	Internal CV AUC	Validation AUC (95% CI)	AUPRC	Acc.	Sens.	Spec.	PPV	NPV	Brier
Clinical	0.76 +/- 0.06	0.730 (0.627–0.830)	0.593	0.69	0.56	0.76	0.54	0.77	0.199
T1 radiomics	0.76 +/- 0.07	0.740 (0.642–0.837)	0.560	0.67	0.85	0.58	0.51	0.88	0.196
T2 radiomics	0.82 +/- 0.05	0.790 (0.679–0.891)	0.750	0.79	0.53	0.92	0.78	0.79	0.159
ADC radiomics	0.84 +/- 0.05	0.810 (0.715–0.895)	0.654	0.73	0.85	0.67	0.57	0.90	0.162
DCE radiomics	0.80 +/- 0.06	0.780 (0.681–0.868)	0.618	0.74	0.68	0.77	0.61	0.82	0.179
Radiomics	0.87 +/- 0.04	0.850 (0.764–0.920)	0.785	0.77	0.65	0.83	0.67	0.82	0.147
ClinRad	0.89 +/- 0.04	0.880 (0.801–0.944)	0.803	0.86	0.79	0.89	0.79	0.89	0.146
Deep fusion	0.91 +/- 0.03	0.910 (0.845–0.959)	0.849	0.84	0.79	0.86	0.75	0.89	0.125

Acc., accuracy; ADC, apparent diffusion coefficient; AUC, area under the receiver operating characteristic curve; AUPRC, area under the precision-recall curve; CI, confidence interval; CV, cross-validation; DCE, dynamic contrast-enhanced magnetic resonance imaging; NPV, negative predictive value; PPV, positive predictive value; Sens., sensitivity; Spec., specificity.

**Figure 2:**
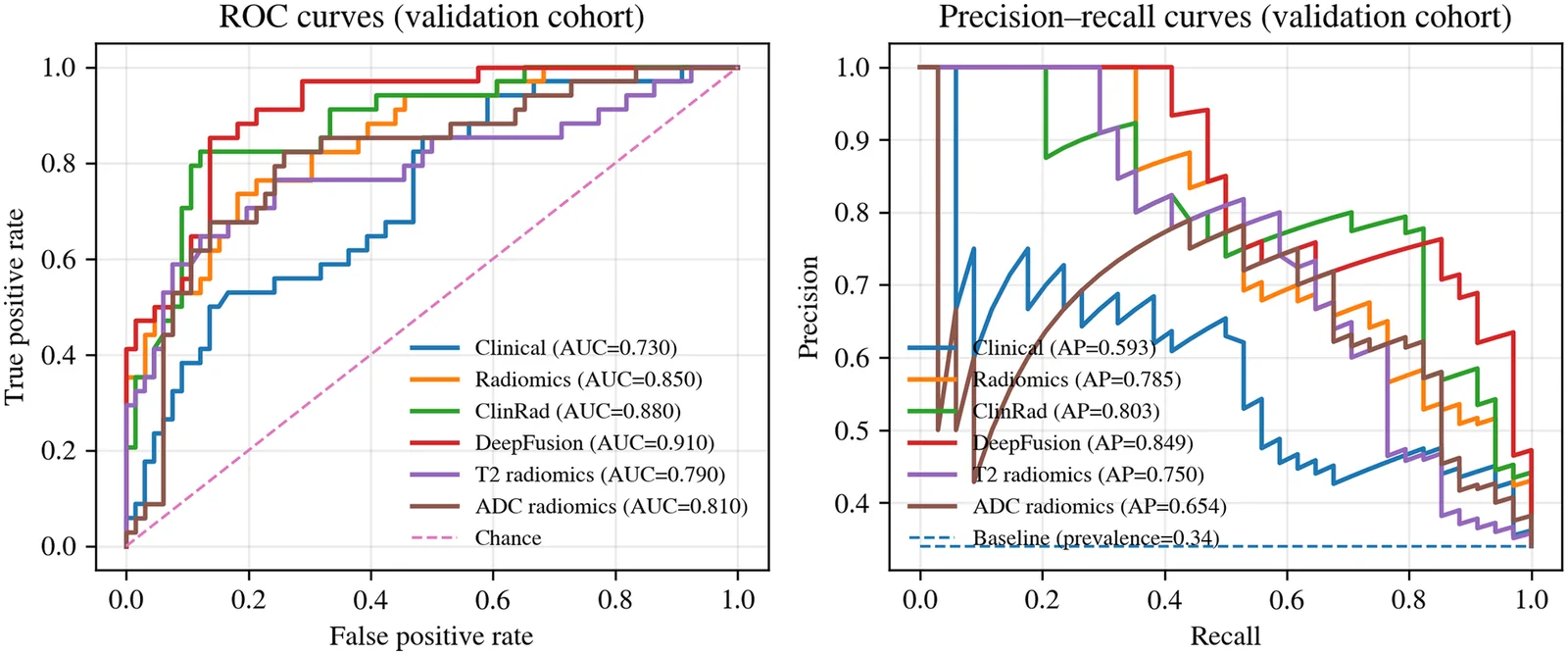
Receiver operating characteristic curves and precision-recall curves for the candidate models in the prospective validation cohort.

At the fixed operating point transferred from the training cohort, the deep fusion model yielded an accuracy of 0.84, sensitivity of 0.79, specificity of 0.86, PPV of 0.75, and NPV of 0.89 in the validation cohort. Compared with the clinical model, the fusion strategy improved sensitivity and PPV while maintaining good specificity, indicating a lower probability of missing metastatic nodes without a disproportionate increase in false-positive classifications.

### Calibration, clinical utility, and AUC comparisons

Calibration results are presented in Table 4 and Figure 3. Although modest calibration drift was observed for several candidate models, the deep fusion model showed the lowest Brier score (0.125), indicating comparatively favorable probabilistic accuracy; however, the calibration intercept (0.463) and slope (0.828) still indicated some degree of miscalibration. The calibration slope and intercept suggested that additional *post hoc* recalibration could further optimize probability estimates in future multicenter implementation studies. Therefore, the model should be interpreted as having strong discrimination, whereas probability estimation still requires further optimization before clinical risk thresholds are used prospectively.

**Table 4:** Calibration metrics in the prospective validation cohort.

Model	Intercept	Slope	ECE (10 bins)	Brier score
Clinical	−0.104	0.582	0.088	0.199
T1 radiomics	0.038	1.361	0.086	0.196
T2 radiomics	0.063	0.701	0.075	0.159
ADC radiomics	−0.071	0.784	0.116	0.162
DCE radiomics	−0.113	0.880	0.124	0.179
Radiomics	0.130	0.847	0.075	0.147
ClinRad	0.400	0.724	0.131	0.146
Deep fusion	0.463	0.828	0.110	0.125

ADC, apparent diffusion coefficient; DCE, dynamic contrast-enhanced magnetic resonance imaging; ECE, expected calibration error.

**Figure 3:**
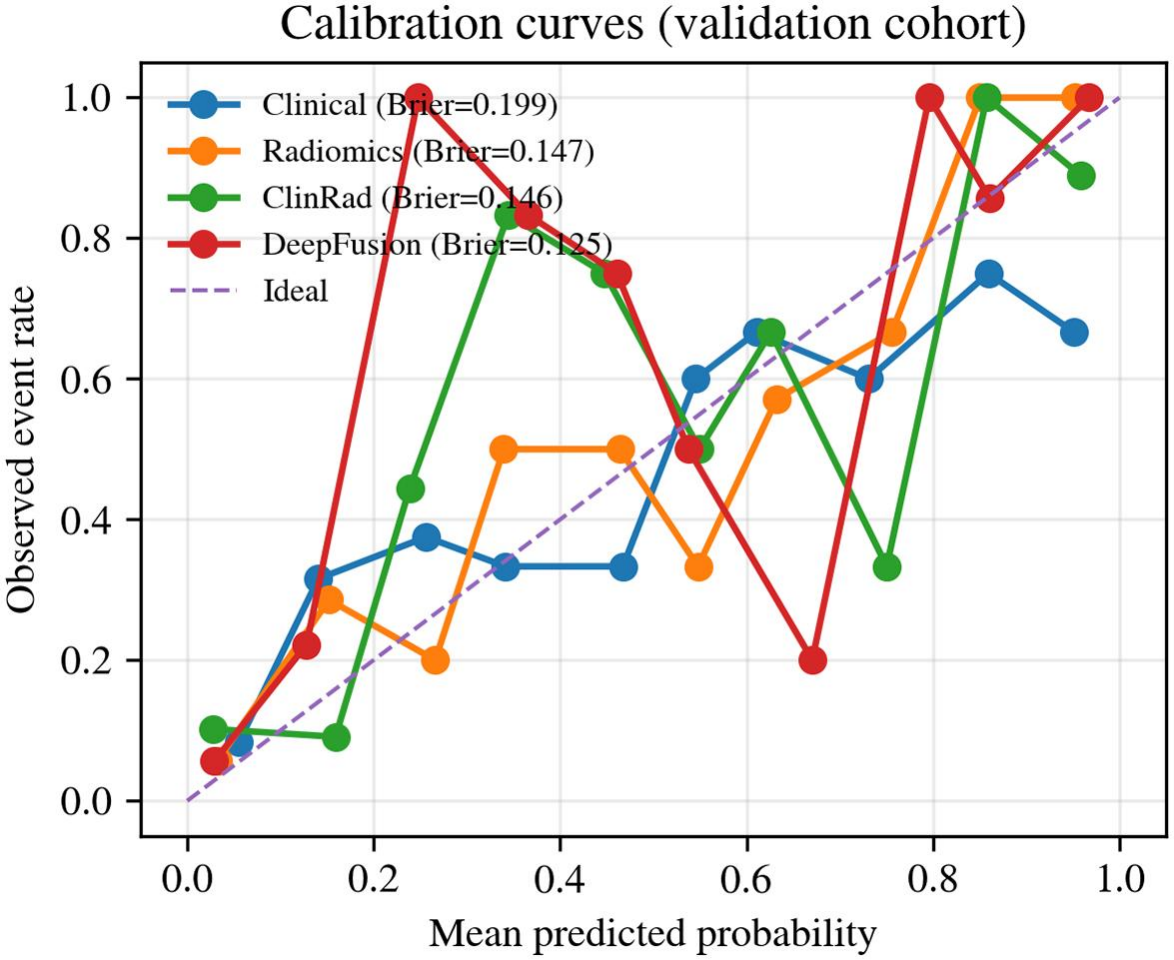
Calibration curves for the clinical, radiomics, clinRad, and deep fusion models in the prospective validation cohort.

Bootstrap-based AUC comparisons confirmed a significant improvement for deep fusion over the clinical baseline (mean delta AUC, 0.180; 95% CI, 0.088–0.280; *P* < 0.001) (Table 5). Decision-curve analysis showed that the deep fusion and ClinRad models provided higher net benefit than both the clinical model and treat-all or treat-none strategies across a broad range of threshold probabilities (Figure 4). The corresponding clinical impact curve indicated a more favorable balance between patients classified as high risk and the expected number of true-positive metastatic cases at clinically relevant thresholds.

**Table 5:** Bootstrap comparisons of AUC between key models in the validation cohort.

Comparison	Mean delta AUC	95% CI low	95% CI high	*P* value
Deep fusion vs. ClinRad	0.030	−0.023	0.085	0.264
Deep fusion vs. Radiomics	0.059	−0.001	0.129	0.059
Deep fusion vs. Clinical	0.180	0.088	0.280	<0.001
ClinRad vs. Radiomics	0.030	−0.039	0.103	0.427
Radiomics vs. Clinical	0.120	0.000	0.237	0.049

AUC, area under the receiver operating characteristic curve; CI, confidence interval.

**Figure 4:**
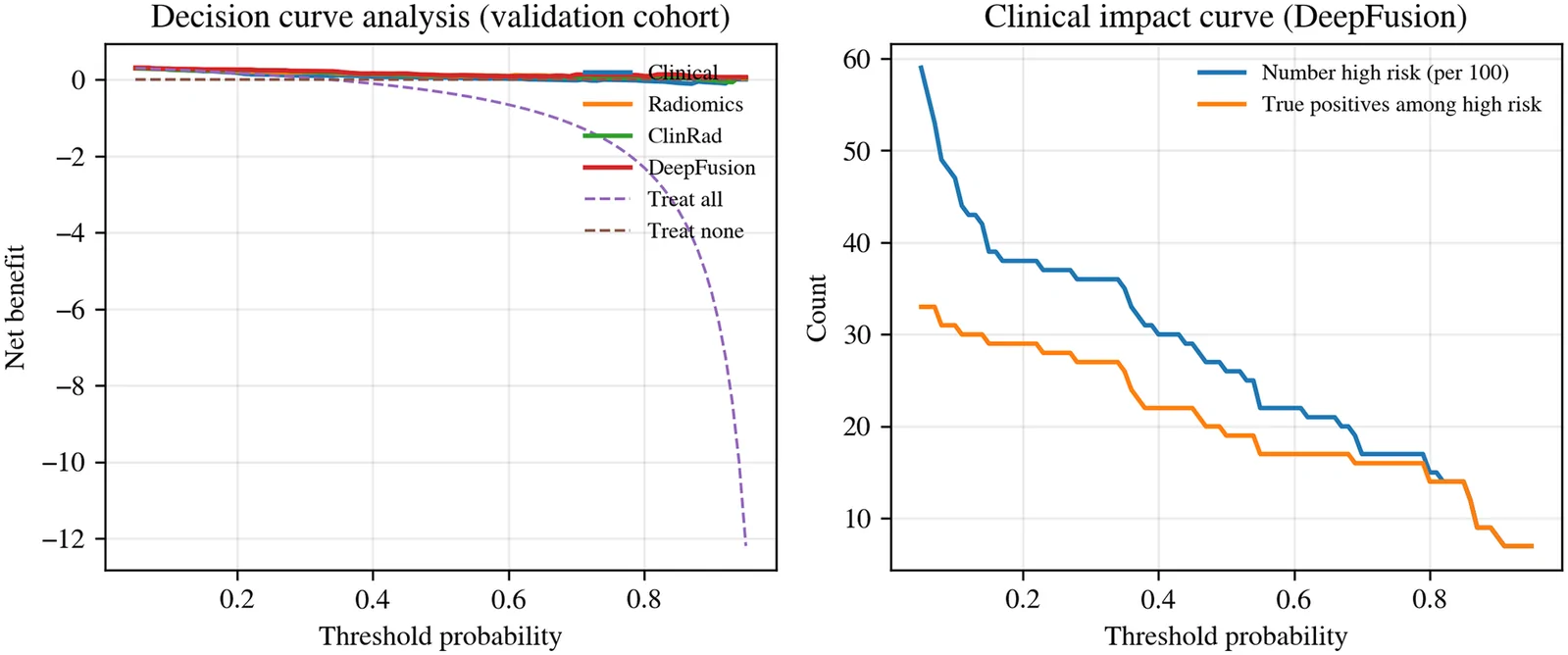
Decision-curve analysis and clinical impact curve in the prospective validation cohort.

### Interpretability and subgroup analyses

The radiomics signature score showed clear separation between patients with and without pelvic LNM in both the development and validation cohorts (Figure 5). Features with the largest standardized coefficients were predominantly texture-based descriptors, implying that spatial complexity, gray-level inhomogeneity, and perfusion-related irregularity were closely associated with nodal spread.

**Figure 5:**
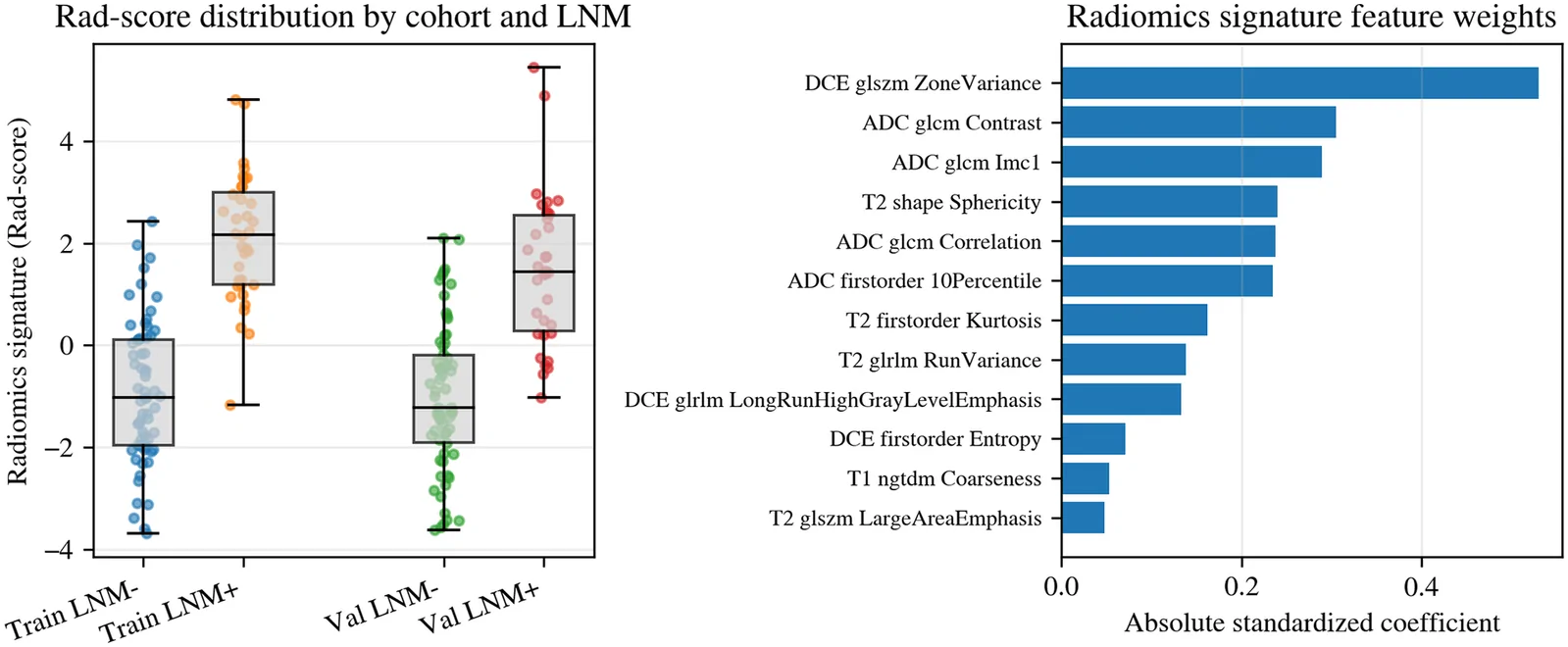
Distribution of the radiomics signature score and standardized weights of the selected radiomics features.

Exploratory subgroup analysis in the prospective cohort showed relatively stable performance of the deep fusion model across FIGO stage strata, histologic subtype, tumor-size groups, and MRI-assessed nodal status (Table 6 and Figure 6). Because several strata were small, especially adenocarcinoma (*n* = 24) and MRI node-positive disease (*n* = 28), these subgroup estimates should be interpreted cautiously. Discrimination remained high in patients with MRI-negative nodes, which is clinically relevant because occult nodal disease is particularly challenging in this subgroup.

**Table 6:** Subgroup AUCs of the deep fusion model in the validation cohort.

Subgroup	*n*	Events	AUC	95% CI low	95% CI high
FIGO <= IB2	58	19	0.949	0.890	0.989
FIGO >= IIA	42	15	0.867	0.740	0.963
Squamous histology	76	26	0.928	0.858	0.980
Adenocarcinoma	24	8	0.844	0.647	0.977
Tumor <= 4 cm	48	14	0.836	0.710	0.940
Tumor > 4 cm	52	20	0.964	0.911	0.997
MRI nodes negative	72	17	0.926	0.861	0.976
MRI nodes positive	28	17	0.893	0.750	0.989

AUC, area under the receiver operating characteristic curve; CI, confidence interval; FIGO, International Federation of Gynecology and Obstetrics; MRI, magnetic resonance imaging.

**Figure 6:**
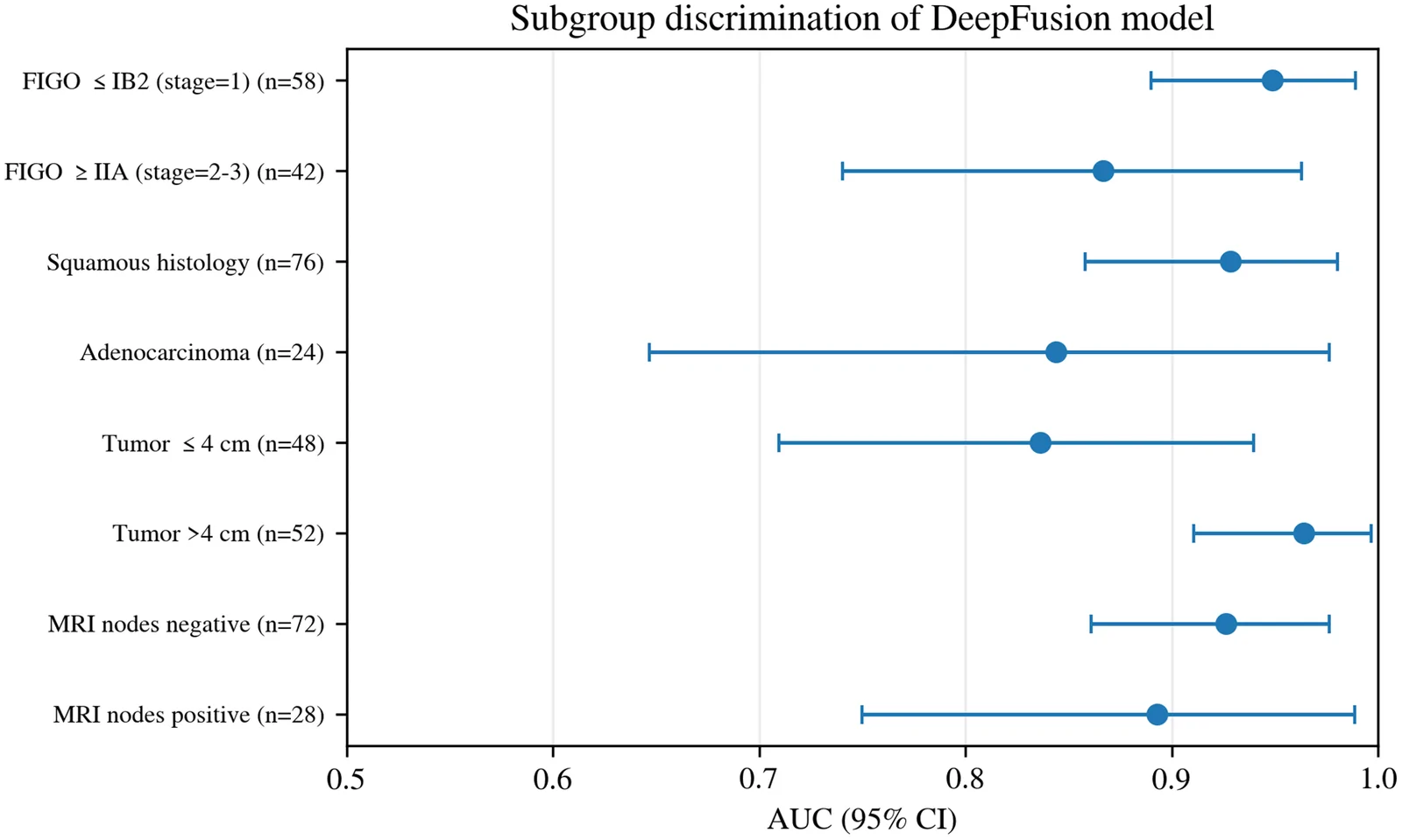
Exploratory forest plot of subgroup area under the receiver operating characteristic curve values with 95% confidence intervals for the deep fusion model in the prospective validation cohort. Subgroup estimates should be interpreted cautiously because some strata were small.

### Incremental value over the clinical model

Reclassification analysis supported the added value of the deep fusion model. Relative to the clinical model, the categorical NRI was 0.535 (95% CI, 0.257–0.791), and the IDI was 0.286 (95% CI, 0.176–0.400) (Table 7). These findings suggest that incorporating quantitative MRI phenotypes can meaningfully improve patient-level risk stratification beyond routine clinical assessment.

**Table 7:** Reclassification improvement of the deep fusion model versus the clinical model.

Metric	Estimate	95% CI
Categorical NRI (cutoffs 0.2/0.4)	0.535	0.257–0.791
IDI	0.286	0.176–0.400

CI, confidence interval; IDI, integrated discrimination improvement; NRI, net reclassification improvement.

## Discussion

This study developed and temporally validated a multi-sequence MRI radiomics framework for pretreatment prediction of pelvic LNM in cervical cancer. Given the single-center design and modest cohort size, these findings should be considered preliminary. The main finding was that the deep fusion model, which combined multi-sequence radiomics with routine clinical variables, consistently outperformed the clinical baseline and showed the most favorable balance of discrimination, probabilistic accuracy, and clinical net benefit in prospective validation. These findings support the concept that quantitative intratumoral imaging phenotypes contain biologically relevant information that is not fully captured by conventional MRI interpretation. These design features define the study's incremental contribution but do not substitute for external multicenter validation or a uniform histopathologic reference standard.

Our results are consistent with prior cervical cancer studies indicating that MRI-derived radiomics features improve nodal metastasis prediction compared with conventional clinicoradiologic variables alone (7–13, 31, 32). Earlier models have often been limited by single-sequence feature extraction, purely retrospective design, or incomplete reporting of calibration and decision-analytic performance. In contrast, the present framework intentionally combined complementary sequence-specific information: T2-weighted imaging contributes anatomy and gross morphology, diffusion and ADC encode tissue cellularity and microstructural restriction, DCE features capture vascularity and perfusion heterogeneity, and clinical variables summarize disease burden. The superior performance of the fusion models over any single-sequence model supports the biological plausibility of this multiparametric strategy. Recent oncologic studies using imaging-derived deep features, multimodal radiomics, and machine-learning workflows also support the value of integrated, interpretable models while emphasizing the need for rigorous validation and clinical-context reporting (33–36).

The observed prominence of texture-based features is also clinically credible. Increased gray-level heterogeneity and irregular zone or run-length distributions may reflect necrosis, stromal remodeling, hypoxia, angiogenesis, and microscopic infiltrative growth, all of which are mechanistically linked to metastatic potential. The addition of clinical variables such as tumor size, FIGO stage, SCC-Ag, and MRI-reported nodal suspicion likely improved performance because these variables summarize disease burden and conventional radiologic risk signals that remain clinically important. From a translational perspective, such a fused model could serve as a noninvasive adjunct at multidisciplinary tumor board discussions, especially when conventional nodal MRI findings are equivocal.

A particular strength of this study is the emphasis on evaluation beyond the AUC. In clinical prediction modeling, high discrimination alone is not sufficient. Calibration determines whether predicted risks correspond to observed event rates, and decision-curve analysis better reflects the clinical consequences of acting on model outputs. We therefore reported calibration intercepts and slopes, Brier scores, AUPRC values, and reclassification statistics in addition to receiver operating characteristic analysis. This broader evaluation strategy is aligned with contemporary methodological guidance for imaging artificial intelligence, radiomics, and prediction-model reporting (14–16, 22). In the present validation cohort, the deep fusion model showed strong discrimination, but calibration metrics did not fully meet ideal values, supporting future recalibration and probability-optimization work.

Several limitations should be acknowledged. First, the study was performed at a single center with a modest sample size, and the validation cohort was temporal rather than external; consequently, the results should be regarded as preliminary and multicenter external validation remains necessary before routine use. The temporal cohort therefore provides a stricter within-center evaluation than random data splitting, but it should not be interpreted as external validation. Second, the reference standard was heterogeneous because some patients were evaluated by surgical histopathology whereas others required composite adjudication; this may introduce verification bias and could affect apparent model performance. Restricting eligibility to patients with surgical histopathology would have excluded 46 non-surgical patients and could have shifted the cohort toward operable disease, creating spectrum and selection bias (22). We therefore retained the composite standard to preserve the intended clinical spectrum while explicitly reporting reference-standard provenance and the prevalence comparison. Nevertheless, because baseline imaging contributed to composite adjudication and MRI-reported nodal suspicion was a candidate clinical predictor, verification and incorporation bias cannot be excluded. The similar observed LNM proportions by reference-standard source do not demonstrate equivalence of the standards. A multicenter study with harmonized MRI acquisition, independent external testing, and pathology-based sensitivity analyses where nodal sampling is clinically appropriate is required. Third, manual segmentation is labor intensive and may restrict scalability, although reproducibility filtering partially mitigated segmentation noise. Fourth, scanner- and protocol-related variability may affect MRI radiomics, and future work should evaluate harmonization strategies such as ComBat when multicenter data become available (37, 38). Fifth, the subgroup analyses were exploratory and limited by small strata, particularly the adenocarcinoma and MRI node-positive subgroups. Finally, although emerging deep-learning and hybrid radiomics studies support the promise of imaging-based nodal prediction, independent validation and prospective clinical impact studies remain necessary before such models can be used to guide treatment decisions (39, 40).

## Conclusion

Multi-sequence MRI radiomics combined with routine clinical variables improved pretreatment prediction of pelvic LNM in cervical cancer compared with a clinical model alone. This approach may support individualized risk stratification and treatment planning after multicenter external validation and workflow optimization.

## Data Availability

The raw data supporting the conclusions of this article will be made available by the authors, without undue reservation. The preprocessing, feature-extraction, model-training, and model-evaluation scripts can be shared for academic research and reproducibility purposes upon reasonable request to the corresponding author, subject to institutional review and removal of any patient-identifying information.
